# MicroRNAs Associated With Colon Cancer: New Potential Prognostic Markers and Targets for Therapy

**DOI:** 10.3389/fbioe.2020.00176

**Published:** 2020-03-10

**Authors:** Junfeng Zhu, Ying Xu, Shanshan Liu, Li Qiao, Jianqiang Sun, Qi Zhao

**Affiliations:** ^1^Department of Clinical Laboratory, Affiliated Hospital of Guilin Medical University, Guilin, China; ^2^Office of Drug Clinical Trials, Affiliated Hospital of Guilin Medical University, Guilin, China; ^3^Department of Clinical Laboratory, General Hospital of Northern Theater Command, Shenyang, China; ^4^School of Automation and Electrical Engineering, Linyi University, Linyi, China; ^5^College of Computer Science, Shenyang Aerospace University, Shenyang, China

**Keywords:** miRNA, hub gene, colon cancer, prognostic marker, bioinformatics

## Abstract

MicroRNAs (miRNAs) are a kind of non-coding RNA (ncRNA) that regulate the expression of target genes and play a role in the occurrence and development of cancers. Colon cancer (COAD) is the second most common cause of cancer-related mortality. However, the prognostic value of miRNAs in COAD is still confusing. In this study, we obtain miRNAs and messenger RNAs (mRNAs) expression profiles of COAD from the Cancer Genome Atlas (TCGA) database. After preliminary data screening and preprocessing, we acquire the expression data of 894 miRNAs and 17,019 mRNAs. Then, compared with the normal samples, 39 upregulated miRNAs and 54 downregulated miRNAs are identified by differential expression analysis. Furthermore, we obtain 1,487 upregulated mRNAs and 2,847 downregulated mRNAs. We confirm nine key miRNAs related to the survival rate of COAD patients. Moreover, by using bioinformatics methods, we get 461 common genes from both the target genes of these nine key miRNAs and differentially expressed mRNAs. Through analyzing the protein-protein interaction (PPI) network of these 461 common genes and survival analysis, we confirm five hub genes as promising biomarkers for COAD prognosis. It is worth mentioning that no previous reports have found that PGR and KCNB1 are related to COAD. We expect these key miRNAs and hub genes will provide a new way for the study of COAD.

## Introduction

Non-coding RNAs (ncRNAs) are a kind of RNA which cannot be translated into protein. NcRNAs were once considered junk RNA. During the last few decades, with the development of high-throughput sequencing technology, people have realized the critical function of ncRNAs (Bussotti et al., 2013). NcRNAs contain functional types of RNA such as transfer RNAs, ribosomal RNAs, microRNAs (miRNAs), long non-coding RNAs (lncRNAs), and so on. Although most of the biological functions are performed by proteins in organisms, ncRNAs also play important roles in various biological processes (Chen et al., 2017, 2019a; Zhou et al., 2017, 2018b; Ferreira and Esteller, 2018). MiRNAs are endogenous, short ncRNAs which can regulate the expression of more than 30% of human genes (Lewis et al., 2005). Abnormal expression of miRNAs is related to various biological modifications, such as apoptosis, cell differentiation, and carcinogenesis (Su et al., 2017; Truong et al., 2017). More and more studies show that miRNAs play a necessary role in the occurrence and development of a variety of cancers, including lung cancer, breast cancer, and colon cancer (Seo et al., 2019).

Colon cancer (COAD) is a common malignant tumor that happens at the junction of rectum and sigmoid colon. In 2018, COAD ranked second for mortality and fourth for incidence (Bray et al., 2018). The 5-year survival rate of colon cancer patients is ~65% (Siegel et al., 2017). Until now, the histological feature is the only prognostic indicator for COAD. According to the histological feature, it is hard to decide to receive adjuvant chemotherapy after surgery or not. Therefore, it is imperative to excavate biomarkers for prognosis of COAD. In 2016, Caritg et al. confirmed three miRNAs (miR-103a-3p, miR-143-5p, and miR-215) as prognostic markers of COAD in patients (Caritg et al., 2016). Then Bobowicz et al. reported that five miRNAs (miR-1296, miR-135b, miR-539, miR-572, and miR-185) have prognostic values for colon cancer in patients (Bobowicz et al., 2016). Later, Maierthaler et al. showed that the miR-122 and miR-200 families have prognostic value in COAD (Maierthaler et al., 2017). However, experimentally determining the prognostic value of miRNAs is not only time-consuming but also costly. So far, many computational methods have been proposed in the field of bioinformatics, for example, circRNA-disease association (Ge et al., 2019; Zhao et al., 2019), drug-side effect (Ding et al., 2019), lncRNA-miRNA interaction (Liu et al., 2020), and lncRNA-protein interaction predictions (Hu et al., 2018; Zhao et al., 2018a,b; Bao et al., 2019; Shen et al., 2019). Therefore, there is an urgent need for developing effective bioinformatics analysis to identify molecular mechanisms related to COAD from the accumulated clinical and experimental data.

TCGA is a large database which contains epigenomic and standardized clinical data from massive samples of each kind of cancer. The data of TCGA includes exon expression data and miRNAs expression data, copy number segments, DNA methylation, phenotype, and so on. Gene chips laid on a high-throughput test can detect thousands of gene expressions in one experiment. Therefore, TCGA database can help us to obtain a large amount of genetic information about cancer in a short time and provide new targets for diagnosis and treatment of cancer (Rajendran et al., 2012, 2018; Rajendran, 2016). Several lines of evidence suggest that some modifications in miRNAs, such as ectopia, mutation, and overexpression, can cause severe pathological alterations (Xiao and Rajewsky, 2009). These effects are attributable to the translation of messenger RNA (mRNA) into protein regulated by miRNA (Bartel, 2018). Besides, different kinds of miRNA present particular expression levels in specific sorts of cancers (Hou et al., 2017). Recently, Xu et al. demonstrated that four miRNAs were significantly associated with the overall survival of COAD patients (Xu et al., 2016). Nevertheless, this study only analyzed the relationship between the expression of miRNAs and COAD patient survival. It did not explore the role of the target genes of these miRNAs in COAD. Hence, the mRNA and miRNA data of COAD from TCGA should be considered together to detect new biomarkers.

In this study, we download miRNA and mRNA expression data of COAD from TCGA database. We do a series of analyses of miRNA and mRNA, such as expression difference analysis, Gene Ontology (GO) analysis, Kyoto Encyclopedia of Genes and Genomes (KEGG) analysis, survival analysis, and PPI network analysis. Then nine key miRNAs (miR-217, miR-144, miR-129, miR-125a, miR-125b, miR-375, miR-328, miR-486, and miR-194) and five hub genes (PPARGC1A, COL1A1, SYT1, PGR, and KCNB1) are confirmed to have prognostic value in COAD. The novel promising prognostic miRNA and mRNA identified in our study will provide a new approach for clinical and experimental research in COAD.

## Materials and Methods

### Data Set, Differential Expression Analysis, and Survival Analysis

The “miRNA mature strand expression RNAseq by IlluminaHiseq,” “gene expression RNAseq,” and “phenotype” dataset of COAD are downloaded from TCGA. The “miRNA mature strand expression RNAseq by IlluminaHiseq” dataset contains the miRNA expression data from 261 samples, including 8 normal samples and 253 COAD samples. Then the “gene expression RNAseq” dataset consists of mRNA expression data from 329 samples, including 43 normal samples and 286 COAD samples. Furthermore, the “phenotype” dataset contains the clinical-pathological data of 545 samples, including 85 normal samples and 460 COAD samples.

We analyze the downloaded dataset as follows: above all, we separate COAD tissue and adjacent non-tumor colon tissue according to the sample number. Then we remove the data of miRNAs and mRNAs with reported expression data for <50% of the patients. Next, a CancerSubtypes package is employed to analyze the expression data of mRNA from the “gene expression RNAseq” dataset. The differentially expressed mRNAs are identified by using the thresholds which are |log2fold-change (FC)| > 1.0 and adjusted *p* < 0.05 for COAD samples compared with the normal samples. Then we identify the differentially expressed miRNA by analyzing the expression data of miRNAs from the “miRNA mature strand expression RNAseq by IlluminaHiseq” dataset in the similar way but using a different threshold which are |log2FC| > 3.0 and adjusted *p* < 0.05. In addition, a volcano map is drawn by ggplot2 package.

We use the Cox regression analysis to investigate the relationship between each miRNA/mRNA expression level and the overall survival rate of COAD patients in the “phenotype” dataset. Log-rank *P* < 0.05 is considered statistically significant for survival differences. Moreover, Kaplan–Meier curves of nine key miRNAs and five hub genes are drawn by the survminer package.

### Prediction of Target Genes of miRNAs and Functional Enrichment Analysis

The target genes of nine key miRNAs are predicted by three kinds of online analysis software including miRDB (http://www.mirdb.org/miRDB/), TargetScanHuman (version 7.2, http://www.targetscan.org/), and mirDIP (http://ophid.utoronto.ca/mirDIP/). Then the Venn diagram is applied to confirm the common genes both in the target genes of miRNA and differentially expressed mRNA. To further understand the biological functions of the common genes, we perform GO and KEGG pathway enrichment analyses by using KOBAS (version 3.0; https://kobas.cbi.pku.edu.cn/anno_iden.php) online tool. *P* < 0.05 is regarded as statistically significant.

### PPI Network Analysis

The STRING (version 11.0, http://string-db.org) is used for searching PPI of the common genes. At the start, a Venn diagram is used to identify the common genes both in the target genes of the nine key miRNAs and the differential expression mRNAs. After importing the official gene symbols of the common genes into STRING, we get the PPI network of the common genes. Then, Cytoscape (version 3.7.1) is applied for the visualization of PPI networks. The confidence score 0.4 is used as the cut-off criterion.

### Confirmation of Hub Genes

CytoHubba, an app of Cytoscape, is applied to confirm hub genes. We employ a Venn diagram to extract the overlapping genes of the top 50 genes by six different algorithms, including MCC, Degree, Closeness, Radiality, Betweenness, and Stress. These overlapping genes are confirmed as the hub genes. Subsequently, we utilize Cox regression analysis to determine the prognostic role of the hub genes.

## Result

### Identification of Differentially Expressed miRNAs and mRNAs in COAD

**Figure 1:**
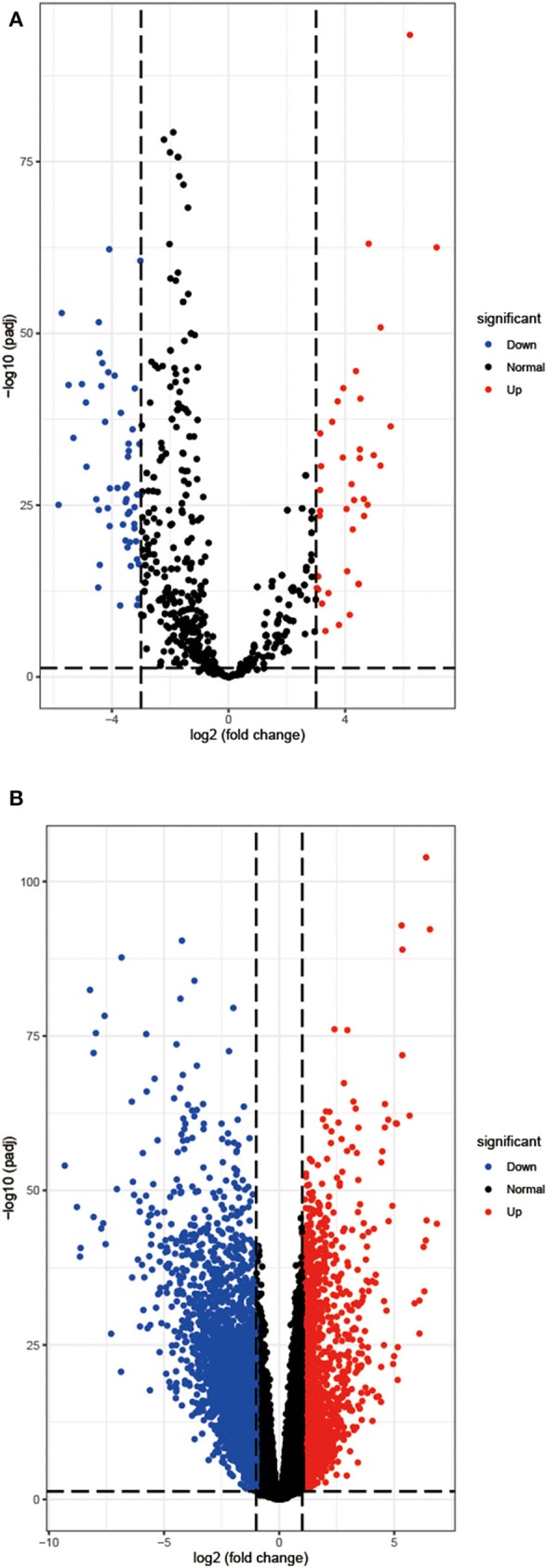
The differentially expressed miRNAs **(A)** and mRNAs **(B)** of COAD. Red, up-regulation; blue, and down-regulation.

Based on the analysis of the CancerSubtypes package, 93 differentially expressed miRNAs are acquired, including 39 upregulated miRNAs and 54 downregulated miRNAs (Figure 1A). Then in a similar way, 4,334 differentially expressed mRNAs containing 1,487 upregulated miRNAs and 2,847 downregulated mRNAs are extracted (Figure 1B).

### Identification of miRNA With Prognostic Value in COAD

**Figure 2:**
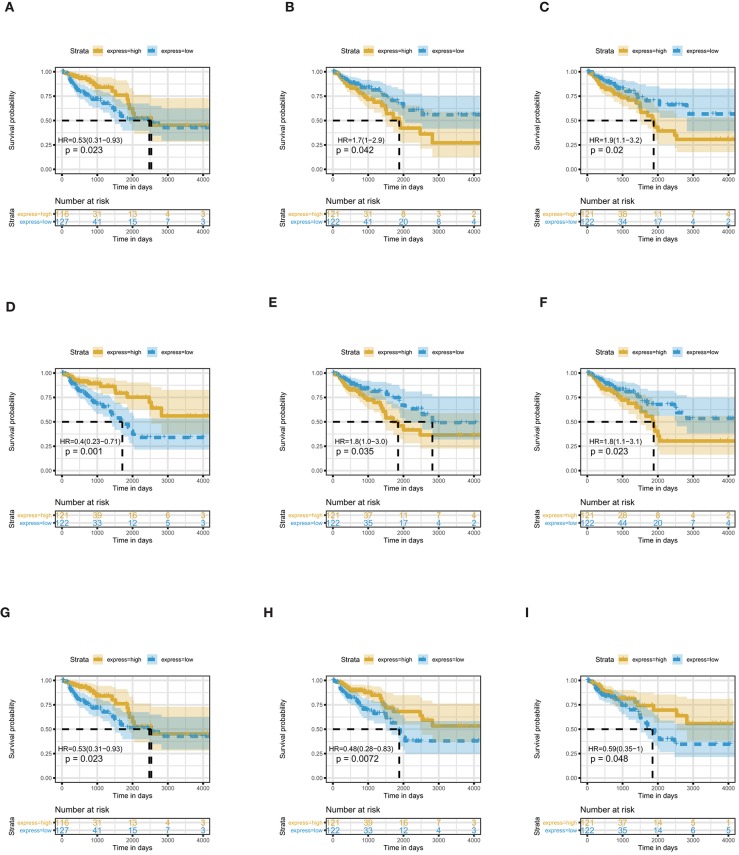
Nine differentially expressed miRNAs are associated with overall survival in COAD patients by using Kaplan–Meier curve, Log-rank test. **(A)** miR-129; **(B)** miR-217; **(C)** miR-125a; **(D)** miR-375; **(E)** miR-328; **(F)** miR-125b; **(G)** miR-144; **(H)** miR-194; **(I)** miR-486.

Through survival analysis, we find nine miRNAs that are significantly associated with the overall survival of COAD patients (Figure 2). The name, Log2FC, *p*-value and adjusted *p*-value of these key miRNAs are displayed in Table 1. In these miRNAs, miR-217 and miR-144 are upregulated, miR-129, miR-125a, miR-125b, miR-375, miR-328, miR-486, and miR-194 are downregulated. In COAD, miR-217 specifically inhibits DKK1, which is an important antagonist of the Wnt signaling pathway to promote apoptosis of colon cells (Jia et al., 2019). By controlling the expression of SMAD4, miR-144 inhibits invasion and migration of colon cancer cells (Sheng et al., 2019). High mobility group box protein 1 (HMGB1) plays a part in immune escape in COAD cells (Zheng and Zhu, 2018). MiR-129, which targets the 3′UTR of HMGB1, is able to repress the development of COAD (Wu et al., 2018). By inhibiting cell proliferation and inducing cell apoptosis, miR-125a acts as a suppressor of COAD (Tong et al., 2015). The level of miR-375 is lower in colorectal cancer tissues than normal human colon tissues. In addition, miR-375 exerts an inhibitory effect on the proliferation of colorectal cancer by targeting the 3′UTR of KLF4 (Mao et al., 2016). Recently, a new study reported that miR-375 has a prognostic value in COAD (Huang and Pan, 2019). By regulating SLC2A1/GLUT1, miR-328 participates in the Warburg effect in COAD (Santasusagna et al., 2018). MiR-486 is related to the molecular mechanisms of several cancers, including cervical cancer (Li et al., 2018a), breast cancer (Li et al., 2019), lung cancer (Tian et al., 2019), esophageal cancer (Lang and Zhao, 2018), ovarian cancer (Ma et al., 2016), and pancreatic cancer (Xia et al., 2019). Kelley et al. nearly demonstrated that the expression of miR-486 associates with early-stage of COAD (Kelley et al., 2018). Moreover, Ren et al. reported that miR-486 plays a prognostic role in COAD (Ren et al., 2016). MiR-194, whose expression is upregulated by p53, inhibits THBS1 expression to promote angiogenesis and facilitate tissue repair in COAD (Sundaram et al., 2011). In a previous study, miR-125b is confirmed as a prognostic biomarker of colorectal cancer (Zhou et al., 2018c). Up to now, the prognostic value of four miRNAs (miR-217, miR-125, miR-129, and miR-194) in COAD has not been previously reported. These miRNAs will become new potential prognostic biomarkers of COAD.

**Table 1:** The name, Log2FC, *P*-value, and Adjusted *p*-value of nine key miRNAs in COAD.

**MiRNA name**	**Log2FC**	***p*-value**	**Adjusted *p*-value**
miR-217	3.21	1.10E-11	2.15E-11
miR-144	4.06	1.69E-16	4.25E-16
miR-129	−4.87	4.62E-32	2.61E-31
miR-125b	−3.08	1.94E-12	4.01E-12
miR-125a	−4.36	4.02E-44	4.65E-43
miR-375	−3.71	2.11E-11	4.04E-11
miR-328	−5.72	4.17E-55	1.09E-53
miR-486	−5.83	2.16E-26	8.98E-26
miR-194	−4.44	1.26E-25	4.99E-25

### Prediction of miRNA-mRNA Interaction and Functional Enrichment Analysis

**Figure 3:**
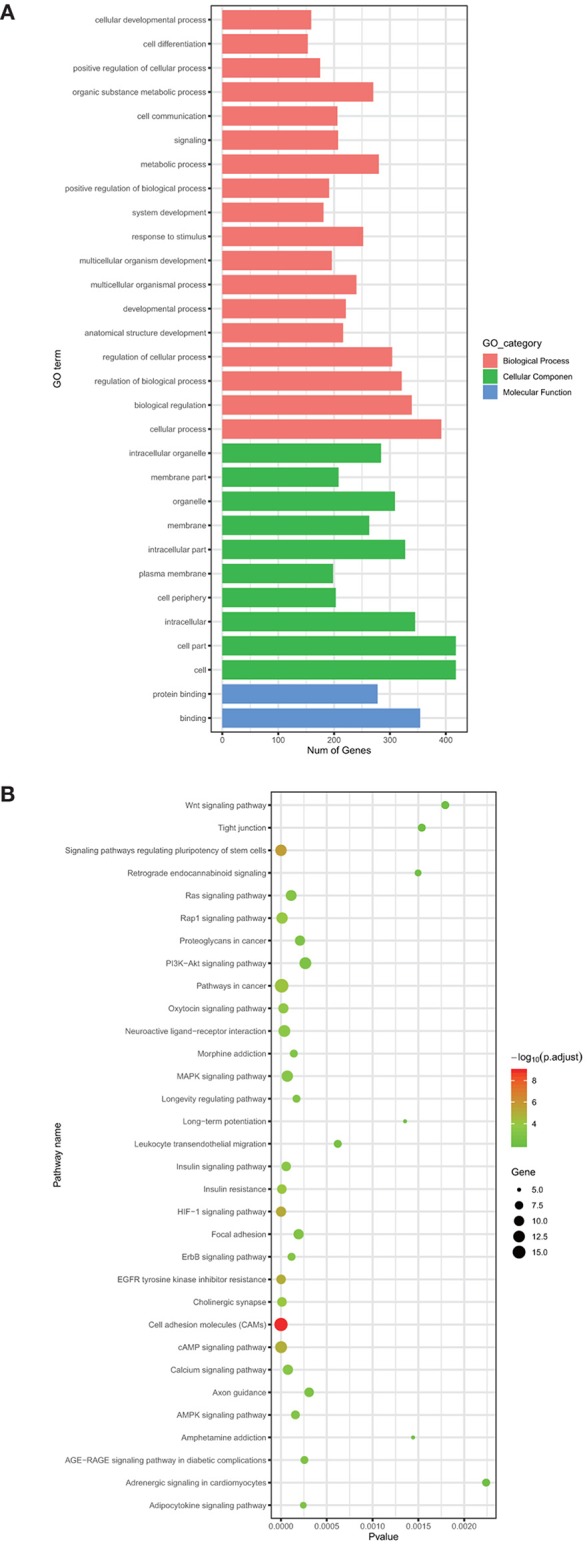
The GO analysis **(A)** and KEGG pathway analysis **(B)** of common genes.

The target gene predictive tools, including miRDB, mirDIP, and TargetScanHuman, are employed to find miRNAs-targeted genes. During this process, 7,592 genes are identified as the target genes of nine miRNAs with prognostic value in COAD. To improve the accuracy of subsequent analysis, a Venn diagram is used to find common genes both in the differentially expressed mRNAs and in the 7,592 target genes. At last, we get 461 common genes. To further understand the biological roles of these 461 common genes, we perform GO analysis and KEGG pathways enrichment analysis of them. The GO analysis shows that 418 genes are involved in cell and cell parts in the cellular component (CC) category. Regarding the biological process (BP) category, this result shows that most of the 461 common genes enrich several functions, including cellular process (392 genes), biological regulation (339 genes), regulation of biological process (321 genes), and regulation of cellular process (304 genes). Then for the molecular function (MF) group, a large proportion of the common genes are mainly enriched in binding and protein binding (Figure 3A). In addition, the KEGG Pathway analysis demonstrates that the 461 common genes enrich cancer-associated signaling pathways, containing pathways in cancer (17 genes, *p*-Value: 7.41E-06), cell adhesion molecules (CAMs) (16 genes, *p*-Value: 7.43E-11), cAMP signaling pathway (13 genes, *p*-Value: 1.22E-06), signaling pathways regulating the pluripotency of stem cells (12 genes, *p*-Value: 2.55E-06), MAPK signaling pathway (12 genes, *p*-Value: 6.88E-05), HIF-1 signaling pathway (10 genes, *p*-Value: 7.70E-07) and proteoglycans in cancer (10 genes, *p*-Value: 2.08E-04) (Figure 3B). Pathways in cancer include Wnt signaling pathway, P53 signaling pathway, and so on. These signaling pathways involve the development of COAD. A previous study showed that CAMs can regulate cancer cell invasion and tumor metastasis (Geletu et al., 2018). The cAMP signaling pathway takes part in regulating various biological processes including cell proliferation, secretion, metabolism and apoptosis. Targeted modulation of cAMP signaling pathway can induce proliferation and apoptosis of a variety of malignant lymphoma cells (Mehta and Patel, 2019).

### Confirmation of Hub Genes

**Figure 4:**
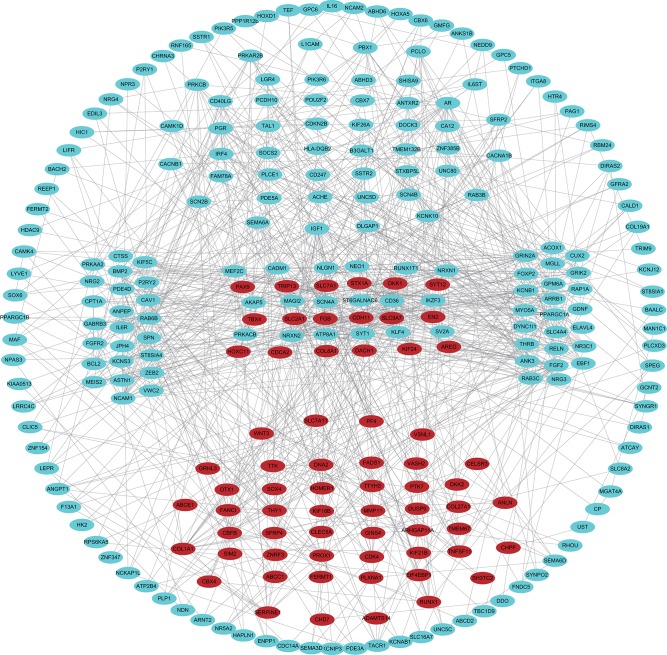
PPI network of common genes. The blue and red dots represent down-regulated and up-regulated genes, respectively.

**Figure 5:**
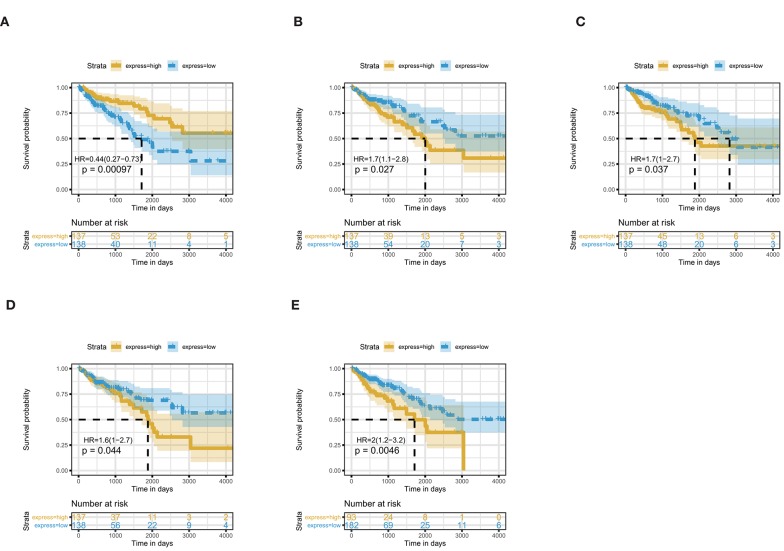
Five hub genes are associated with overall survival in COAD patients by using Kaplan–Meier curve, Log-rank test. **(A)** PPARGC1A; **(B)** COL1A1; **(C)** SYT1; **(D)** PGR; **(E)** KCNB1.

The 461 common genes are used to construct a PPI network which contains 351 nodes and 881 edges (Figure 4). We utilize the cytoHubba app of Cytoscape software to calculate the connectivity between the genes. Because the genes with high connectivity can play an important role in the cancers, we identify five hub genes. The gene symbol, Log2FC, *p*-value, and adjusted *p*-value of these hub genes are showed in Table 2. Furthermore, through survival analysis, we find the five hub genes that have prognostic value in COAD (Figure 5). Among them, when the level of PPARGC1A expression rises, the survival rate of COAD patients improves. Conversely, when the expression levels of COL1A1, SYT1, PGR, and KCNB1 decline, the survival rate of COAD patients improves. As an activator of p53, PPARGC1A can suppress cancer cell apoptosis (Sen et al., 2011). COL1A1 participates in the process of focal adhesion and may influence the metastatic ability of cells (Tian et al., 2015). COL1A1, which is upregulated in COAD, may be a biomarker for colon cancer progression (Yang et al., 2019). Because of down regulation in left-sided colon carcinoma compared with right-sided colon carcinoma, STY1 possibly plays a role in their genetic susceptibilities to neoplastic transformation (Zhu et al., 2013). As the receptor of hormones, PGR is very important for breast growth. Moreover, it is related to the development of breast cancer and endometrial cancer (Kurozumi et al., 2017; He et al., 2019). As a prognostic biomarker for gliomas, KCNB1 suppresses tumor growth by inducing autophagy (Wang et al., 2017). So far, there is no study showing the effect of PGR and KCNB1 in COAD. Therefore, PGR and KCNB1 may be new potential prognostic biomarkers in COAD.

**Table 2:** The Gene symbol, Log2FC, *P*-value, and Adjusted *p*-value of five hub genes in COAD.

**Gene symbol**	**Log2FC**	***P*-value**	**Adjusted *p*-value**
COL1A1	2.21	6.17E-15	2.71E-14
SYT1	−1.06	1.64E-03	2.55E-03
PGR	−2.56	6.35E-24	5.93E-23
KCNB1	−2.56	1.47E-29	2.21E-28
PPARGC1A	−2.11	4.53E-16	2.18E-15

## Discussion

COAD is a common gastrointestinal tumor. High mortality and morbidity are the main clinical features of this disease. At present, the diagnosis of COAD has many problems, such as poor specificity and low time efficiency (Benson et al., 2017; Sun et al., 2019). Previous studies show that some genetic changes are closely associated with the occurrence of COAD (Li et al., 2018b; Wei et al., 2018). For this reason, it is critical to find prognostic biomarkers for the treatment of COAD. It is well-known that the experimental research on the role of miRNAs needs many materials and costs huge time. Compared with traditional experimental methods, the bioinformatics method is cost-effective and timesaving when studying the role of miRNAs. Therefore, in this study we research the prognostic role of miRNAs in COAD using the bioinformatics methods. Furthermore, the role of target genes in COAD can further prove the accuracy of miRNAs predictive results. Hence, we assess not only four miRNAs but also two target genes of nine miRNAs with prognostic value as new potential prognostic markers for COAD. In addition, the target genes with prognostic value can also be therapeutic targets for COAD. These findings will provide a basis for further research on diagnoses of COAD, as well as a new target of monoclonal antibody drugs for treatment of COAD. In future, we plan to use several previous effective computational models to identify colon cancer-related miRNAs (Chen et al., 2018a,b, 2019b).

Despite some promising results, there are several limitations in the current study. First, all data analyzed in the study come from TCGA database, and there is no actual clinical patient data involved in the study. Next, we confirm several novel key miRNAs and hub genes that have never been reported to own prognostic value in COAD. But their molecular mechanisms should be further explored by the experimental way. In the end, more and more studies reported on the role of lncRNA in COAD (Wang et al., 2018; Zhou et al., 2018a). However, lncRNAs are not involved in this study. In the following study, we will collect enough clinical data for further verifying the results of the study. Then we will design a series of experiments to investigate the molecular mechanisms of novel key miRNAs and hub genes. Moreover, lncRNAs should be introduced into the prognostic prediction system for improving accuracy.

## Data Availability Statement

Publicly available datasets were analyzed in this study. This data can be found here: https://www.cancer.gov/about-nci/organization/ccg/research/structural-genomics/tcga.

## Author Contributions

QZ contributed to the design of the study protocol. JZ and YX performed statistical analysis and drawn the pictures. LQ and JS contributed to the writing of the study protocol. SL downloaded data. All authors approved the final version of the manuscript.

### Conflict of Interest

The authors declare that the research was conducted in the absence of any commercial or financial relationships that could be construed as a potential conflict of interest.

## Abbreviations

miRNAs: microRNAs
COAD: colon cancer
TCGA: the Cancer Genome Atlas
PPI: protein-protein interaction
ncRNAs: Non-coding RNAs
HMGB1: High mobility group box protein 1
GO: Gene Ontology
KEGG: Kyoto Encyclopedia of Genes and Genomes
CAMs: cell adhesion molecules
CCL-1: cell adhesion molecule 1
CC: cellular component
BP: biological process
MF: molecular function
MAPK: Mitogen-activated protein kinase
HIF-1: hypoxia-inducible factor-1.
